# Pinpointing Conditions for a Metabolic Origin of Life: Underlying Mechanisms and the Role of Coenzymes

**DOI:** 10.1021/acs.accounts.4c00423

**Published:** 2024-10-05

**Authors:** Joris Zimmermann, Emilie Werner, Shunjiro Sodei, Joseph Moran

**Affiliations:** † University of Strasbourg, CNRS, ISIS UMR 7006, 67000 Strasbourg, France; § Department of Chemistry and Biomolecular Sciences, University of Ottawa, Ottawa, Ontario K1N 6N5, Canada

## Abstract

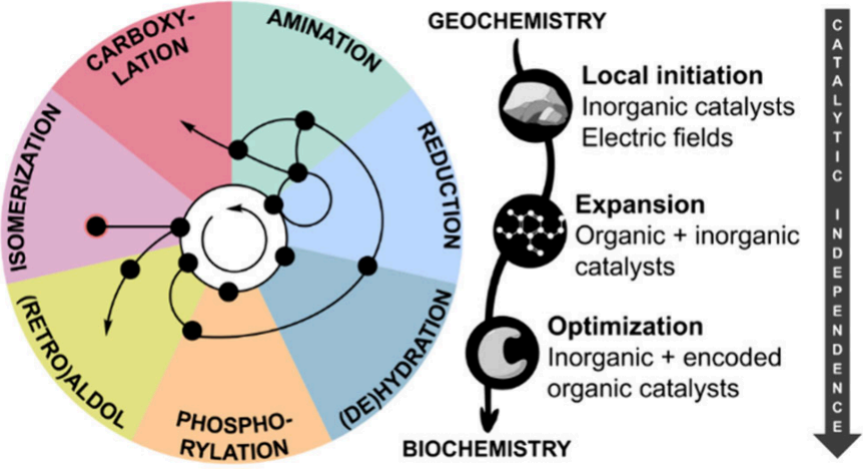

Famously found written on the blackboard of physicist Richard Feynman after his death was the phrase, “What I cannot create, I do not understand.” From this perspective, recreating the origin of life in the lab is a necessary condition for achieving a deep theoretical understanding of biology. The “metabolism-first” hypothesis is one of the leading frameworks for the origin of life. A complex self-organized reaction network is thought to have been driven into existence as a chemical path of least resistance to release free energy in the environment that could otherwise not be dissipated, rerouting energy from planetary processes to organic chemistry. To increase in complexity, the reaction network, initially under catalysis provided by its geochemical environment, must have produced organic catalysts that pruned the existing flux through the network or expanded it in new directions. This boot-strapping process would gradually lessen the dependence on the initial catalytic environment and allow the reaction network to persist using catalysts of its own making. Eventually, this process leads to the seemingly inseparable interdependence at the heart of biology between catalysts (coenzymes, enzymes, genes) and the metabolic pathways that synthesize them. Experimentally, the primary challenge is to recreate the conditions where such a network emerged. However, the near infinite number of microenvironments and sources of energy available on the early Earth or elsewhere poses an enormous combinatorial challenge. To constrain the search, our lab has been surveying conditions where the reactions making up the core of some of the most ancient chemolithoautotrophic metabolisms, which consist of only a small number of repeating chemical mechanisms, occur nonenzymatically. To give a fresh viewpoint in the first part of this account, we have organized the results of our search (along with important results from other laboratories) by reaction mechanism, rather than by pathway. We expect that identifying a common set of conditions for each type of reaction mechanism will help pinpoint the conditions for the emergence of a self-organized reaction network resembling core metabolism. Many of the reaction mechanisms were found to occur in a wide variety of nonenzymatic conditions. Others, such as carboxylate phosphorylation and C–C bond formation from CO_2_, were found to be the most constraining, and thus help narrow the scope of environments where a reaction network could emerge. In the second part of this account, we highlight examples where small molecules produced by metabolism, known as coenzymes, mediate nonenzymatic chemistry of the type needed for the coenzyme’s own synthesis or that turn on new reactivity of interest for expanding a hypothetical protometabolic network. These examples often feature cooperativity between small organic coenzymes and metal ions, recapitulating the transition from inorganic to organic catalysis during the origin of life. Overall, the most interesting conditions are those containing a reducing potential equivalent to H_2_ gas (electrochemical or H_2_ itself), Fe in both reduced and more oxidized forms (possibly with other metals like Ni) and localized strong electric fields. Environments that satisfy these criteria simultaneously will be of prime interest for reconstructing a metabolic origin of life.

## Key References

- VarmaS. J.; MuchowskaK. B.; ChatelainP.; MoranJ.Native Iron Reduces CO_2_ to Intermediates and End-Products of the Acetyl-CoA Pathway. Nat. Ecol. Evol.2018, 2( (6), ), 1019–102410.1038/s41559-018-0542-229686234 PMC5969571.^1^ Placing metallic iron powder in CO_2_-rich water selectively leads to the formation of methanol, formate, acetate, and pyruvate. The products obtained closely resemble those derived from the biological AcCoA pathway and suggests the pathway could have nonenzymatic origins.
- KaurH.; RauscherS. A.; WernerE.; SongY.; YiJ.; KazöneW.; MartinW. F.; TüysüzH.; MoranJ.A Prebiotic Krebs Cycle Analog Generates Amino Acids with H_2_ and NH_3_ over Nickel. Chem2024, 10( (5), ), 1528–154010.1016/j.chempr.2024.02.00138803519 PMC7616004.^2^ A nonenzymatic reductive amination of ketoacids is shown to operate alongside a ketoacid-generating reductive aldol chemistry. This example shows the possibility that multiple nonenzymatic metabolic subnetworks can coexist in one environment.
- WernerE.; PinnaS.; MayerR. J.; MoranJ.Metal/ADP Complexes Promote Phosphorylation of Ribonucleotides. J. Am. Chem. Soc.2023, 145( (39), ), 21630–2163710.1021/jacs.3c0804737750669 .^3^ Aggregates of Fe(III) or Al(III) and ADP promote phosphoryl transfer from acetyl phosphate to nucleoside diphosphates to form the corresponding triphosphates. The ADP/ATP couple may have become central to phosphoryl transfer in biology because they could promote their own synthesis.
- DherbassyQ.; MayerR. J.; MuchowskaK. B.; MoranJ.Metal-Pyridoxal Cooperativity in Nonenzymatic Transamination. J. Am. Chem. Soc.2023, 145( (24), ), 13357–1337010.1021/jacs.3c0354237278531 .^4^ Complexes of Fe(III) or Al(III) and pyridoxal (phosphate) are shown to catalyze transamination between ketoacids and amino acids. The recurring role of these metal ions across diverse reactions with different coenzymes likely implicates them at the origin of metabolism.
- ZimmermannJ.; BasarA. B.; MoranJ.Nonenzymatic Hydration of Phosphoenolpyruvate: General Conditions for Hydration in Protometabolism by Searching Across Pathways. ChemRxiv June 3, 2024, 10.26434/chemrxiv-2024-0sk35PMC1172039939557618.^5^ A set of mild conditions, promoted by Fe oxides such as green rust, apply to all hydration reactions of the Krebs cycle and gluconeogenesis including the hydration of phosphoenolpyruvate to 2-phosphoglycerate, which had not previously been reported under nonenzymatic conditions.

## 1 Introduction

The metabolism-first hypothesis for the origin of life suggests that a single continuous chemical process has supported life’s emergence and evolution, starting with the onset of a metabolism-like self-organized reaction network in geochemistry and arriving at modern biology. Numerous authors have put forward ideas that might be described as metabolism-first over the past seven decades, although they differ in their level of abstraction, and in the proposed details (or lack thereof) regarding their similarity to biological metabolism, their thermodynamic driving force, and the geological environment in which they emerged.^6−11^ Most modern ideas consider the emergence of an initial self-organized chemical reaction network as a response to a buildup of free energy by planetary processes.^12^ The catalytic environment where the reaction network emerges should offer the easiest kinetic path by which the energetic buildup can be released. To put the system on the road to biology, it should ideally do so by coupling energy-releasing and energy-consuming processes toward organic synthesis in such a way as to prefigure the essential biological features of catalysis, metabolism, bioenergetics, compartmentalization, and information processing. After initiating under inorganic catalysis, the reaction network would need to produce organic (or organometallic) catalysts for its own transformations. These catalysts change the flux through the existing network (potentially pruning it) or enable new chemical transformations or mechanisms that were not possible before its emergence, referred to as “enabling conditions”.^13^ Eventually, once the reaction network could persist using only the catalysts it makes itself (a state known as metabolic closure^14^), it can start to exist outside the initial catalytic environment.

**Figure 1:**
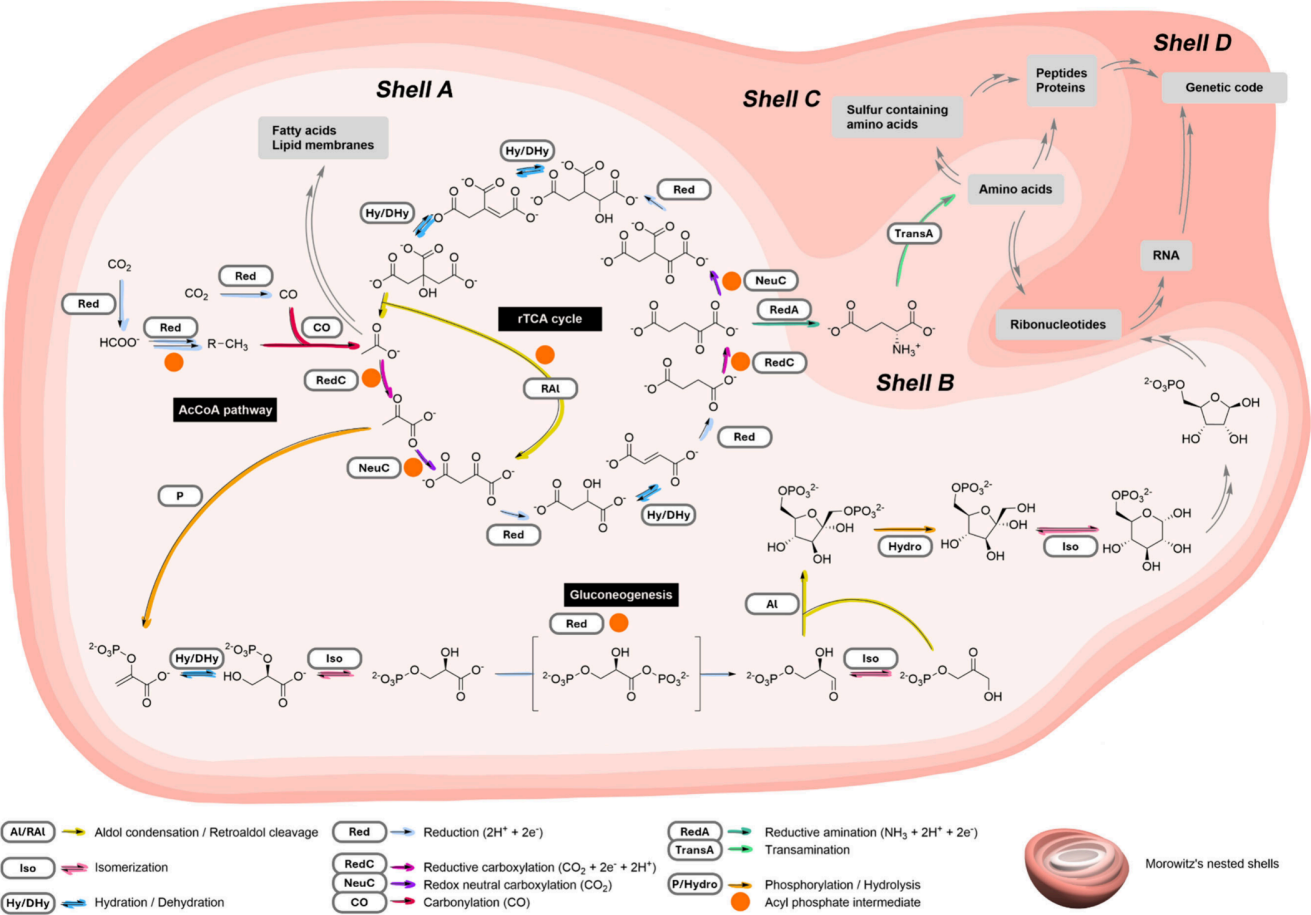
Core metabolism and its transformations.

What would this hypothetical self-organized chemical reaction network have looked like, chemically speaking? Self-organized systems typically result from the simultaneous action of a restrained number of different mechanisms. Interestingly, the core of anabolism is made up of a small set of repeating chemical mechanisms. These mechanisms and their overall organization seem not to have changed since the Last Universal Common Ancestor (LUCA), spanning nearly all of life’s history.^15^ Whatever they are, the reasons that metabolism eluded major changes for four billion years might also have prevented it from drastically changing from its origins in self-organized prebiotic chemistry to the anabolism of LUCA (pruning and metabolic expansion aside). In that case, the ancient pathways at the heart of microbial metabolism could have their roots in the original self-organized prebiotic chemistry. Often invoked in this regard are partial or full versions of the Wood-Ljungdhal pathway (a.k.a. the AcCoA pathway) and the rTCA cycle (a.k.a. the reverse Krebs cycle, a.k.a the reverse citric acid cycle).^11,15−17^ Morowitz represented the development of biochemical networks using hierarchical nested shells (subnetworks) with increasing complexity^8^ (Figure 1). This depiction is not only a graphical presenting choice. Just as the walls of a house are built before the roof, the structure of the metabolic network speaks to the likely emergence of the innermost shells as a necessary precondition for the emergence of the outer shells. The advent of a specific chemical process within one shell acts as a gateway to the emergence of the next outermost shell. The innermost shell, and therefore the first shell thought to have emerged, Shell A, comprises metabolites built from C, H, O, and P. S and N appear in Shell A only in the coenzymes that temporarily activate the metabolites and therefore these elements may not have been required at first. Shell A includes fatty acid synthesis, the AcCoA pathway, the rTCA cycle, and gluconeogenesis. Carbon is fixed within this shell, with C entering as CO_2_ and P entering as phosphate. Shell A is about energy metabolism, amphiphile synthesis and access to the precursors of biosynthesis. Building on the molecules produced in Shell A, Shell B initiates with the entry of N into metabolism in the form of NH_3_ and includes amino acid synthesis. The amino acids produced here, and the peptides derived therefrom, could act as diverse catalysts, prefiguring enzymes. Shell C initiates with the entry of S into metabolism as H_2_S and comprises the diversification of amino acids by incorporation of sulfur to make cysteine and methionine, the former acting as the entry point of S for the rest of metabolism. Finally, Shell D, built from the compounds in Shells A-C, introduces new variations of the mechanisms seen in Shells A-C such as dehydrative *N*-heterocycle synthesis, resulting in the formation of nucleotides and coenzymes. This fourth shell produces molecules involved in genetics, and capable of retroacting on itself and Shells A-C through catalysis (e.g., ribonucleotide-based coenzymes). According to Morowitz, Shells A, B, C and D likely emerged in that order, following the increase in chemical diversity and complexity. The structuring of the metabolic network as a diversity-oriented synthesis radiating from a small core of carbon fixation in Shell A suggests that the molecules of genetics found in Shell D (e.g., nucleotides) were one of many products of a protometabolism. This stands in contrast to the idea that RNA, a polymer of nucleotides, was the progenitor of a protometabolism through its catalytic abilities. Although the precise way Morowitz defined the Shells and ordered their emergence may be questioned (e.g., could parts of Shell B have emerged before some parts of Shell A?), the overall logic of the approach remains appealing.

Toward the goal of experimentally recreating a protometabolism akin to the one described above, our group set out to gather clues to the conditions that might cause such a reaction network to emerge. We approached this challenge by, in a first phase, exploring under which conditions (energetic stresses and chemical and physical constraints) the reactions of core metabolism, especially those in Shells A and B, might occur nonenzymatically. We anticipated that some transformations might occur under a broad range of conditions. Although some of these conditions might have no relevance to the origin of life, they may nonetheless be informative about the underlying nonenzymatic reactivity.^18,19^ Other transformations might be more challenging and highly specific to certain unique environments, helping us to narrow down the conditions where a protometabolism might emerge. The first part of this account will summarize our efforts made in this direction (including the relevant observations of others), organized according to the seven types of transformations identified in Shells A and B (omitting fatty acid synthesis). As depicted in Figure 1, these include: reduction (light blue), hydration/dehydration (dark blue), phosphorylation/hydrolysis (orange), transamination/reductive amination (green), carbonylation/carboxylation (purple), aldol/retro-aldol (yellow), and isomerization (pink).

For each reaction type, we summarize reported prebiotic or nonenzymatic versions of the transformation. Reflecting a second phase of our work, the second part of this account discusses experimental evidence for how the molecules produced in Shell D can mediate pre-existing transformations in the earlier shells or turn on new variants. We conclude by summarizing the conditions thus far found to be suited to overcoming the kinetic barriers presented by the reactions in Shells A and B, which should be useful for guiding future experimental efforts toward creating a nonenzymatic protometabolism.

## 2 Key Reactions in Core Metabolism

**Figure 2:**
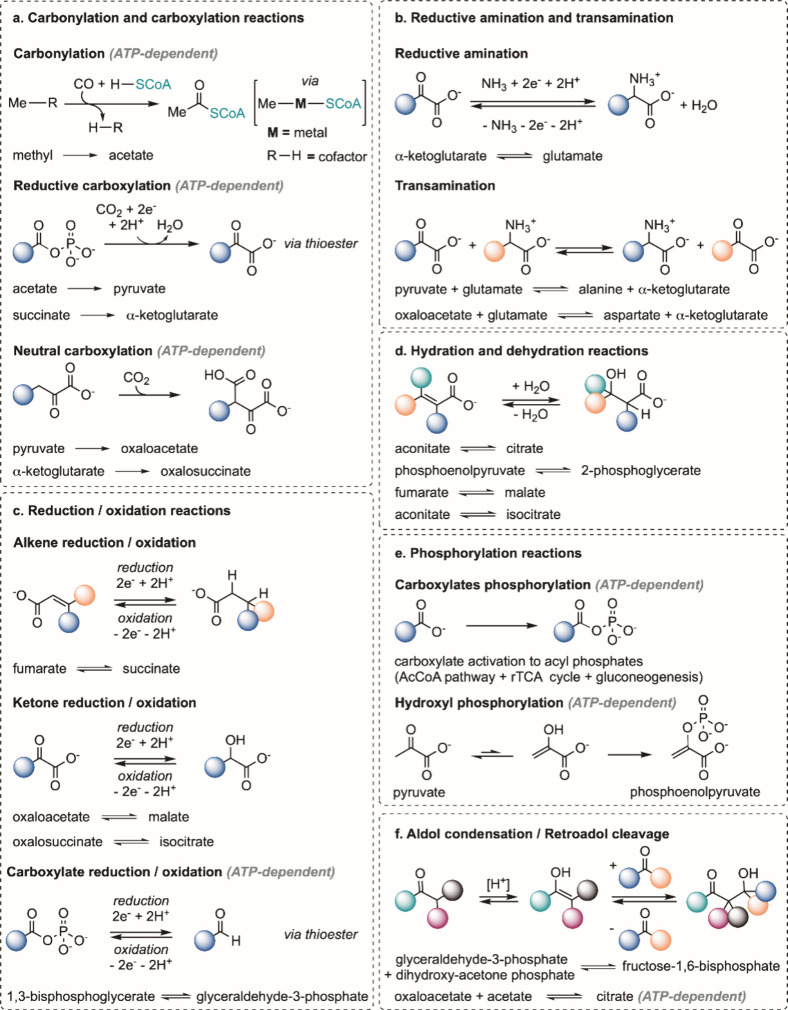
Reaction schemes of the core biological transformations. (a) Carbonylation and carboxylation; (b) reductive amination and transamination; (c) reduction and oxidation; (d) hydration and dehydration; (e) phosphorylation; and (f) aldol condensation and retroaldol cleavage. Different colors represent different R-groups. ATP = adenosine-5′-triphosphate.

This section summarizes experimental reports of the key reactions in core metabolism and what conditions have been shown to permit them in prebiotic chemistry, specifically as they pertain to the biological substrates and reactions shown in Figure 2. We additionally include selected nonenzymatic reports from our group under conditions that are not prebiotically plausible but that we feel yield insight into how these reactions may have operated prebiotically. Unfortunately, analogous reactions on nonbiological substrates, although also informative, could not be included due to space constraints. Summarizing these works in one place brings perspective on the types of environments that might have allowed them all to occur in a cross-compatible way, which we will address at the end of this section and in the general conclusions of the account. As we will see, all major classes of reactions now have nonenzymatic variants, though no single type of condition has thus far been shown to allow all these types of reactions. New search strategies that specifically address this question will be needed moving forward.

### 2.1 Carbonylation/Carboxylation

Arguably the most challenging transformations in Shell A are the C–C bond-forming carbonylation, reductive carboxylation, and redox-neutral carboxylation reactions (Figure 2a).

#### 2.1.1 Carbonylation

The Wood-Ljungdahl pathway gives the acetate scaffold by carbonylation of a metal-bound methyl, followed by reductive elimination forming an acetyl thioester.^20^ To the best of our knowledge, the carbonylation step in isolation has not been studied in a prebiotic context, but it is potentially a mechanistic step in several reports of nonenzymatic chemistry mimicking the Wood-Ljungdahl pathway. Studies show direct access to acetate and pyruvate from CO_2_ and electrochemistry on a Fe_3_S_4_ electrode (cycling between −0.8 and +0.2 V vs NHE at pH 6.5),^21^ native Fe or Ni alone,^1^ H_2_ over Fe_3_O_4,_^22^ H_2_ over Ni_3_Fe,^22^ or Ni_3_Fe on its own.^23^ With H_2_, the reaction is thermodynamically favorable (Δ*G* = −57 kJ/mol) at 100 °C and 25 bar H_2_/CO_2_ (2:3).^24^

#### 2.1.2 Carboxylation

Two types of carboxylation reactions are present in early metabolism, and both are ATP dependent when starting from a carboxylic acid or a ketoacid.^25^ In the rTCA cycle, acetyl-CoA and succinyl-CoA thioesters (which can be produced from the corresponding carboxylic acids by ATP-dependent phosphorylation and substitution with CoA) both undergo a reductive carboxylation to give pyruvate and α-ketoglutarate, respectively. An ATP-dependent redox neutral carboxylation at the α-position of a ketone is also found in the rTCA cycle, transforming pyruvate into oxaloacetate and converting α-ketoglutarate into oxalosuccinate.

The ATP-dependent version of this transformation, which has been proposed to proceed through an enolate of pyruvate and possibly involving a carboxyphosphate intermediate, is exergonic (Δ*G* ∼ −6 kJ/mol), but in the absence of an ATP-driven process the carboxylation is highly endergonic (Δ*G* ∼ +35 kJ/mol),^26^ and occurs spontaneously in the direction of decarboxylation.

Reductive carboxylation of ethylthioacetate was successfully achieved by de Aldecoa et al. electrochemically in the presence of an iron-pyrrhotite-sulfide mixture.^27^ Moreover, the reductive carboxylations of succinate to α-ketoglutarate and of α-ketoglutarate to isocitrate have been found to operate in aqueous microdroplets at room temperature.^28^ The latter transformation presumably occurs through redox neutral carboxylation followed by ketone reduction, though mechanistic evidence is lacking. However, the conversion of pyruvate to oxaloacetate, which could happen through the same mechanism, was not reported. The ability of the aqueous microdroplet to promote this challenging reaction is, at least in part, thought to be due to the strong electric field present at the air–water interface, which lowers the barrier to the reaction.^29^ To date, the direct redox-neutral carboxylation of pyruvate to oxaloacetate and of α-ketoglutarate to oxalosuccinate have not yet been achieved nonenzymatically.

### 2.2 Amination Reactions

Reductive amination and transamination are central processes in amino acid biosynthesis (Figure 2b). Through reductive amination, catalyzed by glutamate dehydrogenase, α-ketoglutarate reacts with NH_4_^+^ and nicotinamide adenine dinucleotide (NAD(P)H) to produce glutamate. Then, through transamination cooperatively catalyzed by aminotransferase and pyridoxal phosphate (PLP), an amino group of glutamate is transferred to a ketoacid from another amino acid to regenerate α-ketoglutarate.

Reductive amination of α-ketoacids with NH_4_^+^ and NADH is not thermodynamically challenging (Δ*G* ∼ −80 kJ/mol)^30^ and nonenzymatic analogs with stoichiometric iron species (such as FeO(OH), FeS, and Fe(OH)_2_) as the reducing agent and in large excess of NH_4_^+^ (above 100 equiv compared to the ketoacid, 375 mM to 1.5 M of ammonia) have been reported.^31−33^ The reduction can also be driven electrochemically with several electrodes at negative potential (−0.74 to −1.46 V vs SCE).^34,35^ Recently, our group demonstrated that reductive amination of ketoacids can be performed at lower ammonia concentrations (6 mM to 150 mM) using H_2_ as reductant and Ni supported on SiO_2_•Al_2_O_3_ or a meteorite as catalyst.^2^ Mayer and Moran carried out a kinetic study on the nonenzymatic reductive amination with BH_3_CN^–^ as a model hydride donor and found that α-ketoglutarate reacts more slowly than other α-ketoacids such as pyruvate and oxaloacetate.^18^ However, nonenzymatic transamination with glutamate is thermodynamically favorable for most α-ketoacids (Δ*G* ∼ −5 kJ/mol)^36^ and happens spontaneously without^37,38^ or with metal ions.^39,40^ Our group found that Cu(II), Ni(II), Co(II), and V(V) are the most active metal ions for nonenzymatic transamination under moderate conditions (pH 7, 20–50 °C) and investigated the detailed reaction mechanism.^41^ Nature’s reason for choosing α-ketoglutarate as the entry point for ammonia in metabolism therefore cannot be due to its inherent reactivity at reductive amination, but rather seems linked to glutamate’s thermochemically favorable position as an amino transfer agent for transamination compared to the other amino acids derived directly from the rTCA cycle.

### 2.3 Reduction/Oxidation

Two types of reduction can be found in core metabolism, namely carbonyl and alkene reduction (Figure 2c). In the earliest stages of prebiotic chemistry, before the emergence of ribonucleosides, the availability of biological organic hydride shuttles (such as NADH) was unlikely. Several theoretical studies suggest hydrogen gas was a likely fuel of protometabolic networks, acting as a source of reduced electrons.^9,10,15^ All of the reduction reactions from core metabolism are thermodynamically favorable (Δ*G* < 0) in H_2_-producing geochemical systems such as hydrothermal vents.^24,42^

Various nonenzymatic analogs of the carbonyl and alkene reductions found in Shell A have been described. The very first step of the Acetyl-CoA pathway is the reduction of CO_2_ to formate. A nonenzymatic version driven by microfluidic pH gradients in the presence of Fe(Ni)S precipitates has been reported by Hudson et al.^43^ This reaction was also identified in the analogs of the Wood-Ljungdahl pathway described in Section 2.1, but also in many prebiotic reaction networks occurring in the presence of CO_2_ or bicarbonate, such as the Ni-catalyzed aldol network under H_2_ of Kaur et al.,^2^ or the sulfite-based photoredox system of Liu et al.^44^ In the latter system, the overreduction of formate to methanol was additionally identified. However, to date, no nonenzymatic reduction of formate to an activated methyl group has been reported in a prebiotic context. In highly acidic conditions, our group found that Fe^0^ was a suitable reducing agent for the reduction of fumarate to succinate and for the reduction of oxaloacetate and oxalosuccinate to malate and isocitrate, respectively.^45^ The reduction of oxaloacetate to malate was also reported using FeS,^46^ or under photochemical conditions using ZnS.^47^ Under electrochemistry (−0.7 V vs SHE) and in the presence of FeS, both reductions of fumarate and oxaloacetate were achieved.^33^ Our group demonstrated the reduction of oxaloacetate and fumarate to malate and succinate, respectively, under H_2_ at room temperature and neutral pH using Ni(0) based catalysts in substoichiometric amounts or using meteorites.^48^ Moving on to the reduction of carboxylates, the reduction of 3-phosphoglycerate (3PGA) to glyceraldehyde-3-phosphate (G3P) in gluconeogenesis, which proceeds through acyl phosphate and CoA thioester intermediates, has yet to be performed nonenzymatically.

Although most redox reactions found in the anabolic reactions of Morowitz’s Shells A and B are reductive, some involve oxidation reactions, including two reactions of the pentose phosphate pathway, the successive transformation from glucose-6-phosphate (G6P) to 6-phosphogluconate to ribulose-5-phosphate. Nonenzymatic conditions have been reported for the latter involving Fe(II) and/or cysteine^49^ at 70 °C under anaerobic acidic conditions.^50^

### 2.4 Hydration/Dehydration

The reversible hydration/dehydration of alkenes occurs in a few steps of the (r)TCA cycle, and in the second step of gluconeogenesis (Figure 2d). The enzymatic dehydration reactions converting malate to fumarate and isocitrate to aconitate are thermodynamically unfavorable (Δ*G* = +3.57 kJ/mol and +2.38 kJ/mol, respectively^30^) and involve an iron sulfur cluster as cofactor. Moreover, the enzymatic hydration of aconitate to citrate is thermodynamically favorable (Δ*G* = −8.49 kJ/mol).^30^ A notable exception, the reversible biological hydration of phosphoenolpyruvate (PEP) to 2-phosphoglycerate (2PGA), is not thermodynamically favorable (Δ*G* = +2.8 kJ/mol)^51^ and does not involve an Fe–S cluster; instead Mg(II) catalysis is needed for the reaction to occur.

Some nonenzymatic versions of these reactions have been reported. The dehydration of malate to fumarate and of isocitrate to aconitate were performed under highly acidic conditions (1 M HCl) at elevated temperatures (70–140 °C) using Zn(II) as promoter.^45^ Similar conditions were reported for the dehydration of isocitrate to aconitate using Zn(II) under concentrated and acidic conditions (0.5 M substrate, pH 5) at 80 °C.^52^ In this study, it was also shown that a wet–dry cycling protocol could dehydrate malate to fumarate at 80 °C. Although other reports show that reduced water activity without wet–dry cycles can drive condensation,^53^ these do not correspond to the reactions covered in this account. The hydration of aconitate to citrate was shown to proceed under highly acidic conditions (1 M HCl) and Cr(III) catalysis at elevated temperatures (140 °C)^45^ or using Zn under the same concentrated and acidic conditions reported above (trace conversion).^52^ The dehydration of malate to fumarate at room temperature was recently described as part of a sequence of the rTCA cycle driven by hydrogen gas and meteorites or metals as catalysts under acidic conditions.^48^ Moreover, aqueous microdroplets were found to dramatically lower the kinetic barriers to the hydration/dehydration reactions of the rTCA cycle, allowing the reactions to equilibrate in microseconds at room temperature, albeit by nebulizing the reaction mixture with a high voltage.^28^

Unlike the other hydration reactions described above, a nonenzymatic analog of the hydration of PEP to 2PGA is unlikely to occur under acidic conditions due to competing hydrolysis of PEP to pyruvate.^54^ Recently, we identified mild aqueous conditions (HCO_3_^–^, pyrophosphate at 25–75 °C, under air) promoted by Fe oxides such as green rust which not only enable the hydration of PEP to 2PGA, but also all the hydration reactions of the rTCA cycle described above.^5^ By providing common conditions for all hydration reactions of the rTCA cycle and gluconeogenesis, this discovery could help narrow down potential conditions for the emergence of a protometabolism.

### 2.5 Isomerization

Found in three steps of gluconeogenesis, reversible isomerization reactions occur through phosphate transfer or enolization. A first instance is the reversible enzymatic conversion of 2PGA to 3-phosphoglycerate (3PGA), proceeding through a phosphorylation-hydrolysis mechanism. The nonenzymatic conversion of 3PGA to 2PGA was reported in 80% conversion in 1 M HCl solution at 100 °C.^55^

The second and third isomerizations, namely the conversions of G3P to dihydroxyacetone phosphate (DHAP) and of fructose-6-phosphate (F6P) to G6P, both proceed through an enediol intermediate, and are both thermodynamically favorable (Δ*G* = −7.1 ± 0.3 kJ/mol,^56^ and Δ*G* = −2.55 ± 0.05 kJ/mol,^57^ respectively). The nonenzymatic isomerization of G3P to DHAP is known and proceeds through the same intermediate via acid–base catalysis, but the intermediate can also undergo an irreversible ß-elimination of the phosphoryl group leading to the decomposition of the triose phosphates to methylglyoxal.^58^ The nonenzymatic reversible conversion of G6P to F6P was studied in alkaline conditions at 100 °C and also proceeds through an enediol intermediate together with irreversible side reactions.^59^

### 2.6 Phosphorylation

Among the phosphorylation reactions appearing in Morowitz’s Shell A (Figure 2e), the most common is the phosphorylation of carboxylates to acyl phosphates^19^ (orange circles, Figure 1). A second type is the phosphorylation of the enolate of pyruvate, which generates PEP, the metabolite with the most energetic phosphate bond (Δ*G* of hydrolysis = −61.9 kJ/mol). Here, pyruvate reacts with ATP to form PEP, AMP and inorganic phosphate. The phosphoryl group introduced at this stage is conserved throughout gluconeogenesis, the pentose phosphate pathway, and *de novo* nucleotide biosynthesis. It is important to note the more challenging energetics of phosphorylation of carboxylates (acyl phosphate hydrolysis Δ*G* ∼ −42 kJ/mol) and of pyruvate (Δ*G* of hydrolysis = −61.9 kJ/mol) as compared to ATP (Δ*G* of hydrolysis ∼ −30 kJ/mol). From these energetic values it is clear that ATP is not able to drive forward phosphorylation because of a high energy P–O bond but rather because the ATP to ADP ratio within the cell is maintained extremely far from equilibrium by ATP synthase and chemiosmosis.^60^ Why would a metabolism be built around an energy currency that is too weak to do its job at 1:1 stoichiometry? It has been suggested that acetyl phosphate formed from the Wood-Ljungdahl pathway may have preceded ATP as an energy currency,^10^ but no nonenzymatic phosphoryl transfer from acetyl phosphate to other carboxylates has been reported thus far. Despite much impressive work on the nonenzymatic phosphorylation of various metabolites,^61−63^ nonenzymatic phosphorylations of the type found in Morowitz’s Shell A are largely absent.

**Figure 3:**
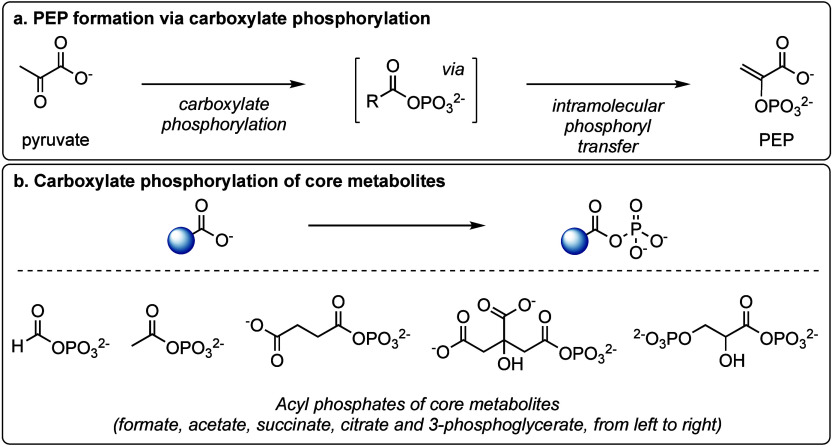
(a) PEP formation via carboxylate phosphorylation. (b) Carboxylate phosphorylation of core metabolites.

We recently reported a mechanistic study showing that PEP is formed nonenzymatically through carboxylate phosphorylation followed by intramolecular phosphoryl transfer (Figure 3a).^19^ This result points to the phosphorylation of carboxylates as a potential single mechanism for the formation of all phosphorylated core metabolites in Shell A (Figure 3b), at least until the formation of phosphoribosyl pyrophosphate (PRPP), strengthening arguments for the emergence of metabolism based on chemical self-organization from a small number of chemical mechanisms. However, it must be noted that the conditions employed were not likely on the early Earth (i.e., using solid P_4_O_10_ in paste conditions), highlighting the importance of developing nonenzymatic methods for carboxylate phosphorylation under prebiotic conditions. If carboxylate phosphorylation was as central to a self-organized protometabolic reaction network as it is to microbial metabolism, it must have emerged in an environment where carboxylate phosphorylation was particularly easy. Identifying such environments will greatly constrain the search for metabolic emergence and should be a priority for future research.

### 2.7 Aldol/Retroaldol

The aldol reaction (Figure 2f) occurs in gluconeogenesis, when G3P condenses on its isomer, DHAP, to give the aldol product fructose-1,6-bisphosphate (F1,6BP). Ralser et al. have found a prebiotically plausible version of this aldol reaction under freezing conditions (−20 °C).^64^ Due to the irreversible decomposition of triose phosphates at pH > 6,^58^ the reaction remains challenging at room temperature.

One retroaldol reaction occurs in the rTCA cycle (Figure 2f). In the biochemical pathway, the carboxylate function of citrate is first phosphorylated to give citryl phosphate, and then converted to the CoA thioester, which facilitates the retro-aldol cleavage. This ATP-dependent transformation is nearly thermoneutral, with Δ*G* ∼ +2 kJ/mol.^42^ An ATP independent enzymatic version of this transformation is also known.^65^ A nonenzymatic retroaldol reaction of citrate was first observed within aqueous microdroplets, presumably due to the electric field exerted at the air–water interface.^28^ Recently, our group has shown that this reaction happens under the same conditions reported to enable the hydration of core metabolites, in a mixture of iron oxides, pyrophosphate, and air.^5^

As shown in Section 2.1.2, the ATP-dependent reductive and redox-neutral carboxylations are among the most challenging in Shell A. To interconvert ketoacids by forming C–C bonds in an alternative way, prebiotic chemistries based on aldol condensations were developed by our group and others by taking into account the apparently central role of glyoxylate and pyruvate in metabolic subnetworks that do not depend on ATP.^39,66,67^ More recently, our group found that, at 23 °C, reduction by H_2_ with a Ni catalyst drives a related reductive aldol network starting from glyoxylate and oxaloacetate to similarly form intermediates of the rTCA cycle while enabling their reductive amination to amino acids under the same conditions.^2^ To what extent this alternative “metabolism-adjacent” chemistry is relevant to the origin of life is unclear. However, the latter example shows the possibility that multiple nonenzymatic metabolic subnetworks can coexist in one environment.

### 2.8 Summary

Nonenzymatic analogs of the specific reaction classes found in Shells A and B, except for carboxylate phosphorylation, have now been demonstrated for at least one of the biological substrates in Figure 1. These early works are largely exploratory in nature and the conditions identified were often very different from each other. Few of these works have focused on mutual compatibility between reactions. In future work, a systematic investigation by reaction types spanning multiple pathways, rather than focusing on the different pathways individually, should increase our chances to triangulate potential conditions conducive for the emergence of a protometabolism.

## 3 Involvement of Coenzymes in Nonenzymatic Pathways

### 3.1 Nonenzymatic Catalysis by Coenzymes

**Figure 4:**
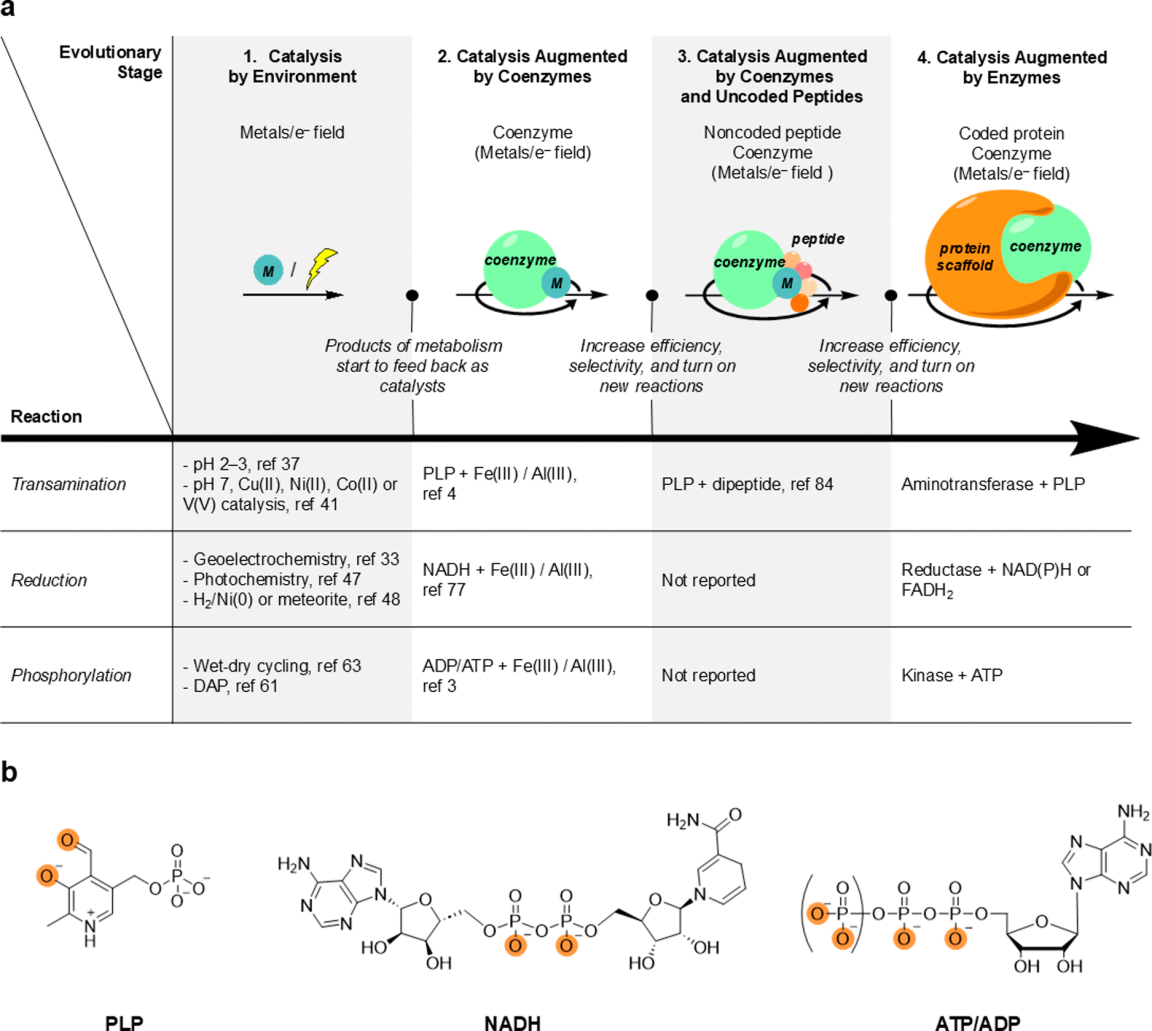
(a) Proposed evolutionary trajectory of catalysis from protometabolism to metabolism. (b) Structure of the studied coenzymes. Experimentally supported binding sites for metals are highlighted in orange.

From Section 2, we saw that all the reaction classes in Shell A (except for carboxylate phosphorylation) have now been demonstrated nonenzymatically, though not necessarily all under the same conditions nor for every substrate on which they occur in metabolism. However, to complexify toward a life-like system, the self-organized reaction network also needs to be able to produce molecules that can catalyze some of its existing reactions, which could divert reaction flux preferentially through some channels over others, potentially pruning the network. Conversely, as the network produces molecules with increased complexity, some of these may be able to react in new ways that were not previously possible, enabling the extension of the network.^13^ These molecules might also have new catalytic properties, turning on new reactions elsewhere in the network. Indirect evidence that such a process occurred on route to modern metabolism might still be seen in the structure of metabolism itself, and in the use of coenzymes as cocatalysts in many biological reactions. In modern metabolism, approximately 60% of enzymes rely on supportive entities called cofactors. These can be metals, metal ions, or small organic molecules known as coenzymes. Coenzymes have a variety of organic structures and thus a wide range of catalytic functions. They are widely used in over 30% of present enzymes to promote core metabolic reactions. Their origins are considered to date back prior to the emergence of life.^68^ Bioinformatic analyses suggested that NAD and ATP were central molecules within the metabolism of LUCA.^69^ PLP was also found to be a good candidate as an early constituent of metabolism due to its relatively simple structure and biosynthetic pathway.^68^ These reports suggest an early introduction of coenzymes into protometabolism, likely acting as prebiotic catalysts and playing a pivotal role in elaborating early metabolic networks before genetically encoded enzymes. Recently, their catalytic activity without the respective protein scaffolds has been investigated from the perspective of a nonenzymatic origin of metabolism (Figure 4).

### 3.2 Transamination: PLP and Metals

Beyond the simple metal catalysis of transamination discussed in the previous section, it was reported that pyridoxal, a vitaminic derivative of PLP, could enhance the catalysis of metal ions under mild reaction conditions. Harrison et al. reported the synthesis of aspartic acid and related amino acids through transamination with Cu(II) and pyridoxal or pyridoxamine, albeit without catalytic turnover.^70^ Our group showed that the dual presence of pyridoxal and Fe(III) caused an increase in initial reaction rate of up to 180-fold compared to Fe(III) alone and 90-fold compare to pyridoxal catalysis alone, clearly showing a cooperative effect of the coenzyme and the metal.^4^ Unlike most reported metal-promoted reactions in prebiotic chemistry,^71^ this system embodied a widely accepted minimal criteria for catalysis by having a turnover number higher than 1 (up to 7.9). Among the panel of metal ions surveyed, this cooperative effect was most pronounced for Fe(III) and Al(III). As those two are among the most abundant metals in the Earth’s crust, this observation strongly suggests the possibility of these transamination conditions on the prebiotic Earth. Furthermore, the nonenzymatic reaction mechanism with metals and pyridoxal was found to be like that of modern metabolic systems, smoothly filling the gap between prebiotic and biotic transamination.

### 3.3 Nonenzymatic Reduction by NADH with and without Metals

In metabolism-first models, the prebiotic production of NADH and its insertion into the reaction network as an electron and proton shuttle is a likely key step in the self-complexification toward modern metabolism. Nonenzymatic electron transfer reactions with NADH^72^ as well as the reduction of NAD^+^ to NADH^73−76^ have been reported in a prebiotic context. However, its major role as hydride donor is less explored. Recently, the reducing ability of NADH has been evaluated in a prebiotic context. Our group reported that the nonenzymatic reduction of α-ketoacids to α-hydroxyacids with NADH was enabled by various metal ions.^77^ We also found that the transformation occurred in an enantioselective manner (up to 60% ee), induced by the formation of a metal-coenzyme complex. Intriguingly, similar to the case of pyridoxal-catalyzed transamination, the most effective metal ions were found to be Fe(III) and Al(III). Additionally, Nogal et al. reported the nonenzymatic reductive amination of ketoacids with ammonia mediated by NADH in the absence of metal ions.^78^

### 3.4 ADP-Metal Complexes as a Phosphoryl Transfer Catalyst

ATP plays a central role in biochemistry by transferring phosphoryl groups mainly produced by chemiosmosis to other metabolites, which in turn powers all energy-demanding processes in the cell. Adenosine-5′-diphosphate (ADP) is usually, but not always, the byproduct of phosphoryl transfer. However, it is unclear why the ADP/ATP pair was chosen for this role, rather than the other canonical or noncanonical nucleotides. One hypothesis is that adenine derivatives were chemically best suited to promoting phosphoryl transfer.^9^ Interestingly, previous work has shown that ATP can be obtained from ADP by nonenzymatic phosphoryl transfer from acetyl phosphate in the presence of Fe(III) in water and at room temperature.^79,80^ Other canonical nucleoside diphosphates failed to be phosphorylated under these conditions. Our group has shown that ADP promotes regioselective nonenzymatic phosphoryl transfer from acetyl phosphate to other ribonucleotides and ribonucleosides at the 5′-position at room temperature in water, again in the presence of Fe(III) or Al(III).^3^ No other nucleoside diphosphates were found to promote the reaction under the tested conditions. The phosphorylation of nucleoside diphosphates (NDPs) to nucleoside triphosphates (NTPs) was the most efficient of the various phosphoryl transfers studied. This system may represent an intermediary evolutionary stage between nonenzymatic substrate-level^61,63,81^ and enzymatic phosphorylation. The initial production of ADP by the metabolic network might have “turned on” the conversion of NDPs to NTPs, allowing the metabolic network to expand in a way that was not possible before the existence of ADP.

### 3.5 Toward Enzyme–Coenzyme Cooperativity

Early protometabolism would likely have been producing short uncoded peptides, which have already been shown to form under prebiotic conditions.^82^ These short peptides could have acted as catalysts or cocatalysts with coenzymes,^83^ foreshadowing the coenzyme-enzyme cooperativity seen in biology today.

One recent attempt to reveal the cooperativity of short peptides and coenzymes was reported by Yu et al.^84^ They carried out transamination in the presence of pyridoxal and dipeptides and found that dipeptides bearing a proline residue have a positive effect on the reaction yields. Furthermore, the reported amino acid products were enantioenriched due to the stereochemistry of the dipeptides, possibly by forming an intermediate complex of a pyridoxal and a dipeptide. Although catalytic turnover with respect to the dipeptide or coenzyme was not demonstrated, this report contributes to the understanding of how peptides might assist coenzyme catalysis, naturally leading to more efficient catalysis by their larger coded enzyme cousins.

## 4 Conclusions

Toward the goal of identifying the conditions under which a nonenzymatic protometabolism first emerged, our group has systematically searched for conditions under which the reactions underpinning the core of anabolism occur nonenzymatically, especially for the reactions in Shells A and B as described by Morowitz. Our results, and those of other groups, reveal that some reactions are easy and occur under a variety of conditions. H_2_ seems to be an excellent chemical reductant, but reductive electrochemistry is also effective. Other reaction types are more challenging and were found to occur only under very particular conditions, narrowing the search. Some considerations from the latter category: 1) CO_2_ fixation reactions mimicking the AcCoA pathway depend on reduced Fe, Ni or Co; H_2_ or geoelectricity may generate these in situ; 2) many other reactions surveyed within Shell A are promoted by acid/base catalysis or by strong electric fields; 3) reactions like carboxylation and reductive carboxylation seem to operate only in environments that offer catalysis by strong electric fields; 4) several reactions in which coenzymes are able to nonenzymatically promote the types of reactions needed for their own biosynthesis or turn on new reactions appear to depend on Fe(III) or Al(III).

Taken together, environments having a redox gradient allowing the possibility to produce, at least transiently, reduced Fe and Fe (III) at the same time, possessing a proton gradient, and possessing strong electric fields would seem to be the ideal environment to allow the reactions in Shell A and B to emerge nonenzymatically as a true network. Considering that chemiosmosis, quantitatively the most important driver of the cell’s bioenergetics, is based on harnessing proton gradients, an environment bearing proton gradients may also be useful in addressing its origins.^85^ Alkaline hydrothermal vents are environments that could fill these requirements as they present natural cavities, pH gradients, and electric fields.^10,86,87^ Of course, this analysis is based on the available experimental evidence and new findings could change these conclusions. Ongoing work from our lab aims to experimentally test specific environments that might simultaneously embody these features. Last, future efforts will also focus on selectivity, a central feature of metabolism, specifically on finding conditions where core metabolism-like reactions occur simultaneously without cross-inhibition and are favored over side pathways.

## ABBREVIATIONS

LUCA: last universal common ancestor
AcCoA: acetyl-CoA
rTCA: reverse tricarboxylic acid
RNA: ribonucleic acid
DNA: DNA
ATP: adenosine-5′-triphosphate
NAD(P)H: nicotinamide adenine dinucleotide (phosphate) reduced form
PLP: pyridoxal phosphate
SCE: saturated calomel electrode
SHE: standard hydrogen electrode
3PGA: 3-phosphoglycerate
G3P: glyceraldehyde-3-phosphate
G6P: glucose-6-phosphate
2PGA: 2-phosphoglycerate
PEP: phosphoenolpyruvate
DHAP: dihydroxyacetone phosphate
F6P: fructose-6-phosphate
AMP: adenosine-5′-monophosphate
ADP: adenosine-5′-diphosphate
F1,6BP: fructose-1,6-bisphosphate
FADH_2_: flavin adenine dinucleotide reduced form
NDP: nucleoside diphosphate
NTP: nucleoside triphosphate
